# Do CA125 response criteria overestimate tumour response in second-line treatment of epithelial ovarian carcinoma?

**DOI:** 10.1038/sj.bjc.6601501

**Published:** 2004-01-20

**Authors:** B Gronlund, H H Hansen, C Høgdall, E V S Høgdall, S A Engelholm

**Affiliations:** 1 Department of Oncology, Rigshospitalet, Copenhagen University Hospital, 9 Blegdamsvej, DK-2100 Copenhagen, Denmark; 2 Department of Gynecology, Rigshospitalet, Copenhagen University Hospital, DK-2100 Copenhagen, Denmark; 3 Department of Clinical Biochemistry, Statens Serum Institute, DK-2300 Copenhagen, Denmark

**Keywords:** ovarian neoplasms, recurrence, second-line treatment, chemotherapy, CA125, response

## Abstract

Recent studies indicate that cancer antigen 125 (CA125) response criteria tend to overestimate a tumour reduction measured by standard WHO response criteria in recurrent epithelial ovarian carcinoma. The aim of the study was to validate the recently introduced GCIG (The Gynaecological Cancer Intergroup) CA125 response criteria in predicting a tumour response measured by WHO (World Health Organization) criteria. Changes in CA125 levels (GCIG criteria) were retrospectively compared with alterations in the tumour load (WHO criteria) during second-line chemotherapy with topotecan or paclitaxel–platinum in 124 consecutive patients with recurrent or refractory disease. In patients assessable by both response criteria (*n*=72), the overall response rate using GCIG CA125 criteria was 57% (95% confidence interval (CI): 45–69%) and significantly higher than the response rate of 39% (95% CI: 28–51%) using WHO response criteria (*P*=0.045). The GCIG CA125 criteria had a sensitivity of 96% (95% CI: 82–100%), a specificity of 68% (95% CI: 52–81%) and an accuracy of 79% (95% CI: 68–88%) in predicting a response measured by WHO criteria. In conclusion, the GCIG CA125 response criteria seem to overestimate a tumour response by WHO criteria when monitoring the efficacy of second-line chemotherapy with topotecan or paclitaxel–platinum in patients with epithelial ovarian carcinoma.

Recurrent epithelial ovarian carcinoma is generally considered as an incurable disease and second-line chemotherapy may be administered for palliation of symptoms and extension of survival (Ozols, 1997; Adams *et al*, 1998). Response assessment in recurrent disease is an elusive matter. Many patients presenting with, for example, peritoneal carcinosis and ill-defined tumours cannot be adequately monitored with physical examination and conventional imaging techniques such as computed tomography or ultrasound scans. The biochemical tumour marker cancer antigen 125 (CA125) is a glycoprotein expressed on the cell surface of epithelial ovarian cancer cells. Cancer antigen 125 levels are elevated (⩾35 U ml^−1^) in 82% of newly diagnosed ovarian carcinoma patients and in 74% of patients, the CA125 level is above 65 U ml^−1^ (Bast *et al*, 1983; Tuxen *et al*, 1995). In first-line treatment, serial changes in CA125 concentrations are well correlated with response and survival (Rustin *et al*, 1999). The measurement of CA125 has as such an established role in the monitoring of the efficacy of first-line chemotherapy in both clinical trials and in the individual patient (Berek *et al*, 1999; Meyer and Rustin, 2000).

In contrast, the clinical use of CA125 in monitoring second-line treatment is still controversial (Pearl *et al*, 1994; Davelaar *et al*, 1996; Bridgewater *et al*, 1999). Several studies identify patients in whom the effects of second-line chemotherapy on the CA125 level are in discordance with the radiographic evaluation of changes in the tumour load (Eisenhauer *et al*, 1994; Morgan *et al*, 1995; Davelaar *et al*, 1996). In a recent meta-analysis of 19 phase II trials including 14 different antineoplastic agents and different first-line regimens, it was found that CA125 response criteria tend to overestimate a tumour reduction when compared to WHO (World Health Organization) tumour response criteria (Rustin *et al*, 2000). However, a routine application of a correction factor between the CA125 and the WHO response rates was not recommended.

The primary aim of the study was to validate the recently introduced GCIG (The Gynaecological Cancer Intergroup) simplified CA125 response criteria (Rustin, 2003) in predicting a response by WHO criteria, in the monitoring of second-line chemotherapy in a well-defined group of consecutive patients with epithelial ovarian carcinoma all pretreated with paclitaxel–platinum as first-line chemotherapy. The second aim was to evaluate whether the application of an alternative CA125 ratio response algorithm could increase the accuracy in the prediction of a tumour response.

## MATERIAL AND METHODS

### Patients

Since 1994, when paclitaxel–platinum was introduced as standard first-line chemotherapy for epithelial ovarian carcinoma at the Finsen Center, all patients with ovarian tumours have been consecutively registered. The clinical data from patients with recurrent epithelial ovarian carcinoma have been included in a clinical database (CODOVA: Copenhagen Database for Ovarian Carcinoma; The Danish Data Protection Agency No. 2000-41-0126) (Gronlund *et al*, 2001; Gronlund *et al*, 2002a, 2002b). In the period August 1994 to January 2001, 487 consecutive patients with primary epithelial ovarian cancer received first-line chemotherapy with paclitaxel–platinum. Of these, 124 patients received standardised second-line chemotherapy consisting of topotecan (*n*=64) or paclitaxel–carboplatin (*n*=60) because of refractory, persistent or recurrent disease and these patients constitute the study group. The patient age at the start of second-line chemotherapy was median 59.4 years (range 34.8–77.2 years). A median number of six second-line cycles (range 1–16 cycles) were applied as salvage treatment. The efficacy of the second-line chemotherapy was routinely assessed by imaging techniques (abdominal and endovaginal ultrasonography or CT scans) after every two courses of chemotherapy. Ultrasonographies were performed by senior consultants at the Department of Radiology, Rigshospitalet. The duration of treatment was dependent on evaluation of response and followed departmental guidelines for standard second-line therapy. In patients obtaining a complete response (CR), chemotherapy was continued for two cycles after a CR was obtained. In patients with a partial response (PR) or stable disease (SD), standard antineoplastic therapy was continued until tumour progression. Patients with tumour progression or unacceptable toxicity were offered various regimens such as inclusion in phase II protocols with investigational drugs, endocrine therapy, or they received exclusively supportive care. The duration of third- or fourth-line treatment was similar as for the second-line treatment.

The patient data were retrospectively evaluated with special focus on response to second-line antineoplastic treatment.

### WHO response assessment

The tumour burden was evaluated before the start of second-line chemotherapy (cycle 1) and, thereafter by every second cycle of chemotherapy, that is, in relation to cycles 3, 5, 7, etc. All patients were retrospectively assigned a best response to treatment, that is, CR>PR>SD>progressive disease (PD) using standard WHO criteria (Miller *et al*, 1981). Briefly, a response has occurred if there has been a reduction of at least 50% in the product of the diameters of all measurable lesions determined by two observations not less than 4 weeks apart. Patients having bidimensional disease assessed by ultrasonography (>20 mm) or by CT scan (>10 mm) were defined as having measurable disease. Nonmeasurable (NM) disease was defined as lesions measuring less than 20 mm by ultrasonography or less than 10 mm by CT scan. To obtain a response by WHO criteria, the patients were thus requested to have at least two cycles of second-line chemotherapy followed by evaluation after another 3 weeks, thus precluding patients who succumbed to disease before 6 weeks after the start of second-line treatment. These patients were categorised as having NM disease in this analysis.

### CA125 assay

All patients had blood samples taken routinely before the start of second-line treatment and then prior to each cycle of chemotherapy. Serum levels of CA125 were determined using an enzyme immunoassay (Abbott CA125 EIA, Abbott Laboratories, Chicago, IL, USA) according to the manufacturer's instruction. The within-assay coefficient of variation (CV) was 6.6% (*n*=60), whereas the between-assay CV was 6.2% (*n*=10) at a control sample of 30 U ml^−1^. Cancer antigen 125 levels below 35 U ml^−1^ were considered as normal in agreement with the literature (Bast *et al*, 1983; Kenemans *et al*, 1993; Markman, 1994), and were regardless of the exact CA125 result changed to <35 U ml^−1^.

### CA125 response algorithms

Two different CA125 response algorithms were applied (1) the GCIG CA125 response criteria and (2) the CA125 ratio criteria (Table 1

**Table 1:** Differences between the GCIG CA125 criteria and the CA125 ratio criteria used in the comparison with the WHO response criteria

	**GCIG criteria**	**Ratio criteria**
Pretreatment samples required (*n*)	2	1
Moment of response evaluation	Variable^a^	Fixed^b^
Confirmation sample required	+	−

Confirmation of GCIG=The Gynaecological Cancer Intergroup; CA125=cancer antigen 125; WHO=World Health Organization.

a Dependent on the time of best response allocation by the WHO response criteria.

b After 1, 2, 3,…,*M* cycles of second-line chemotherapy.

).

#### GCIG CA125 criteria

On the day of the WHO response allocation, the level of the tumour marker CA125 was compared to the pretreatment level and a CA125 response was calculated according to the CA125 response criteria proposed by the GCIG (Rustin, 2003). Briefly, two pretreatment samples at least twice (⩾70 U ml^−1^) the upper cut-off of normal (>35 U ml^−1^) and at least two additional samples after the start of treatment are required to have evaluable disease. A response has occurred if there is at least a 50% decrease that is confirmed by the fourth sample.

#### CA125 ratio criteria

A CA125 ratio was calculated after every series of second-line chemotherapy by dividing the actual CA125 level following treatment, by the pretreatment CA125 value directly prior to the start of second-line treatment, that is, the CA125 ratio after one cycle means the CA125 ratio after one cycle of chemotherapy, and this blood sample was obtained approximately 3 weeks after the first cycle just before the administration of the second cycle (Table 1). Three response categories are defined as proposed by Davelaar *et al* (1996).

Response (CR+PR): ratio ⩽0.5.

SD: ratio >0.5 and <2.0.

PD: ratio ⩾2.0.

Patients with an elevated CA125 level over or equal to 70 U ml^−1^ before the start of second-line treatment were defined as having evaluable disease. Nonevaluable disease was defined as a CA125 level ⩾35 U ml^−1^ and <70 U ml^−1^. To be evaluable by the ratio criteria, only two samples were required. Whereas the GCIG criteria are based on alterations in the numeric values of CA125, the ratio criteria include a time factor, thus reflecting the rate of decline in CA125 levels. Furthermore, the CA125 ratio does not necessarily reflect the CA125 level at the day of the WHO best response allocation.

### Statistical methods

In patients assessable both by WHO criteria (measurable disease) and by CA125 criteria (evaluable disease), the relationship between a CA125 and a WHO response was expressed by means of the following definitions. *Concordance* means that the designations of response or nonresponse following second-line treatment were similar using CA125 and WHO tumour response criteria. The *sensitivity* of the CA125 criteria to depict a WHO response was defined as the number of patients with a concordant response in relation to the total number of patients with a WHO response. The *specificity* of the CA125 criteria was defined as the number of concordant nonresponders in relation to the total number of nonresponders by the WHO criteria. The *positive predictive value* (PPV) of the CA125 criteria was defined as the number of concordant responders in relation to the total number of patients with a CA125 response. The *negative predictive value* (NPV) was defined as the number of concordant nonresponders in relation the total number of nonresponding patients by the CA125 criteria. The *accuracy* of the CA125 criteria was defined as the number of patients with concordant response designations in relation to the total number of patients. The sensitivity, specificity, PPV, NPV and accuracy in predicting a WHO response were calculated for (A) the GCIG CA125 response criteria and (B) the different CA125 ratio response criteria (after 1, 2, 3 and 4 cycles of chemotherapy). The differences in the ability of the various CA125 response algorithms to detect a tumour response by WHO criteria were tested with Fisher's test. *P*<0.05 was considered statistically significant.

## RESULTS

### Tumour response: WHO criteria

Out of 124 patients, 24 had NM disease for assessment by WHO criteria leaving 100 patients eligible for WHO tumour response evaluation. The frequencies of CR and PR were 27% (95% confidence interval (CI): 18–37%) and 14% (95% CI: 8–22%), respectively. The frequencies of SD and PD were 41% (95% CI: 31–51%) and 18% (95% CI: 11–27%), respectively.

### CA125 response: GCIG criteria

Nonevaluable disease was noted in 23 patients with (A) pretreatment levels ⩾35 U ml^−1^ and <70 U ml^−1^ (*n*=8) or (B) only one pretreatment sample (>70 U ml^−1^) (*n*=9), or (C) two pretreatment samples (>70 U ml^−1^) and less than four total samples (*n*=1), or (D) a combination of these categories (*n*=5). In the nine patients with only one pretreatment sample, seven patients had a subsequent reduction of more than 75% of the pretreatment CA125 level. Another 14 patients had CA125 levels below the cut-off limit of 35 U ml^−1^, and they were described as CA125 normal.

Hence, 87 patients were eligible for response assessment. The frequencies of CR and PR were 41% (95% CI: 31–52%) and 16% (95% CI: 9–26%), respectively. The frequencies of SD and PD were 31% (95% CI: 22–42%) and 11% (95% CI: 6–20%), respectively.

### Comparison of WHO and GCIG CA125 criteria

Table 2

**Table 2:** Correlation between WHO tumour response criteria and the GCIG CA125 response criteria in second-line treatment of epithelial ovarian carcinoma (*n*=124)

	**WHO criteria**					
**CA125 criteria**	**CR**	**PR**	**SD**	**PD**	**NM**	**Total**
Responders	16	11	14	0	9	50
Nonresponders	0	1	20	10	6	37
NE	7	1	2	7	6	23
Normal^a^	4	1	5	1	3	14
Total	27	14	41	18	24	124

The table depicts the number of patients who fulfilled the criteria. WHO=World Health Organization; GCIG=The Gynaecological Cancer Intergroup; CA125=cancer antigen 125; CR=complete response; PR=partial response; SD=stable disease; NM=nonmeasurable disease; NE=nonevaluable disease.

a CA125 normal: CA125 levels below 35 U ml^−1^.

shows the relationship between WHO criteria and the GCIG CA125 criteria in the monitoring of second-line therapy of epithelial ovarian carcinoma (*n*=124). Of 124 patients, 72 were assessable both by WHO criteria (measurable disease) and by CA125 criteria (evaluable disease). In these patients (*n*=72), the overall response rate using GCIG CA125 criteria was 57% (95% CI: 45–69%) (41/72) and significantly higher compared to the response rate of 39% (95% CI: 28–51%) (28/72) using WHO response criteria (*P*=0.045). Hence, either the GCIG CA125 criteria overestimate the WHO response rate by a factor 1.46 or, alternatively, WHO response criteria underestimate a GCIG CA125 response by a factor 0.68. In an intent-to-treat analysis adding patients with two elevated pretreatment samples and less than four total samples (*n*=1), the response rate (GCIG CA125) was reduced to 56% (95% CI: 44–68%) (41/73), and the difference between WHO and CA125 response rates only tended to significance (*P*=0.055).

The CA125 criteria had a sensitivity of 100% (95% CI: 74–100%) (16/16), a specificity of 55% (95% CI: 42–69%) (31/56), a PPV of 39% (95% CI: 24–56%) (16/41) and an NPV of 100% (95% CI: 89–100%) (31/31) in predicting a CR by the WHO criteria. The CA125 criteria had a sensitivity of 96% (95% CI: 82–100%) (27/28), a specificity of 68% (95% CI: 52–81%) (30/44), a PPV of 66% (95% CI: 49–80%) (27/41), and an NPV of 97% (95% CI: 83–100%) (30/31) in predicting a response (CR+PR) by the WHO criteria (Table 3

**Table 3:** Sequential response rates according to the CA125 ratio criteria (*n*=102)

	**Ratio after one cycle**	**Ratio after two cycles**	**Ratio after three cycles**	**Ratio after four cycles**
CR+PR	32 (23–42)	58 (47–68)	63 (53–73)	64 (53–74)
SD	64 (54–73)	35 (30–46)	28 (19–38)	26 (17–37)
PD	4 (1–10)	6 (2–14)	9 (4–17)	10 (5–19)
Evaluable patients (*n*)	102	93	90	88

CA125=cancer antigen 125; CR=complete response; PR=partial response; SD=stable disease; PD=progressive disease. Numbers in percentage (95% confidence intervals). CA125 ratio was calculated as the CA125 value after *M* (*M*=1–4) cycles divided by the baseline CA125 value.

).

In patients assessable by both criteria (*n*=72), the two response criteria were concordant in 79% (95% CI: 68–88%) (57/72) of patients. Disconcordance in the designation of WHO response and CA125 response was observed in 21% (95% CI: 12–32%) (15/72) of patients. The failure of CA125 response criteria to reflect a tumour shrinkage assessed by WHO criteria was observed in 4% (95% CI: 0.1–18%) (1/28) of the patients. A 50% reduction in the CA125 level despite clinical disease progression (PD) by WHO criteria was observed in 0% (95% CI: 0.0–31%) (0/10) of patients.

A numeric greater proportion of all patients treated with second-line treatment (*n*=124) are assessable by WHO response criteria (81%: 100 of 124 patients) than CA125 response criteria (70%: 87 of 124 patients) (*P*=0.076).

### CA125 response: ratio criteria

Of 124 patients, 102 patients were evaluable for response by CA125 ratio criteria (CA125⩾70 U ml^−1^, one sample), eight patients had nonevaluable disease (CA125: ⩾35 and <70 U ml^−1^, one sample) and 14 patients were described as CA125 normal (CA125 <35 U ml^−1^). As only one pretreatment CA125 measurement is required for eligibility by CA125 ratio criteria, the number of evaluable patients by ratio criteria (*n*=102) is numerically higher than the number of evaluable patients by GCIG CA125 criteria (*n*=87), where two pretreatment measurements are required (Table 1). Table 3 demonstrates the sequential response rates over treatment time according to CA125 ratio criteria. The response rate (CR+PR) after the first cycle of chemotherapy (32%; 95% CI: 23–42%) was significantly inferior to the response rate after the second (58%; 95% CI: 47–68%; *P*=0.0005), third (63%; 95% CI: 53–73%; *P*<0.0001) and fourth cycle (64%; 95% CI: 53–74%; *P*<0.0001) of treatment. The rate of PD increased numerically with the number of cycles of second-line therapy from 4% (after one cycle) to 10% (after four cycles). Of 54 patients (58%) obtaining an initial biochemical response after two cycles, five patients (9%) experienced a subsequent progression in CA125 levels after three or four cycles.

### Comparison of WHO and CA125 ratio criteria

Of 124 patients, 84 were assessable both by WHO criteria and by the CA125 ratio criteria. Table 4

**Table 4:** Sensitivity, specificity, positive predictive value (PPV), negative predictive value (NPV) and accuracy of the CA125 ratio criteria in predicting tumour response (CR+PR) determined by the WHO criteria (*n*=84)

	**CA125 reduction**			
	**Ratio after one cycle**	**Ratio after two cycles**	**Ratio after three cycles**	**Ratio after four cycles**
Sensitivity	51 (34–69)	83 (66–93)	91 (77–98)	97 (85–100)
Specificity	84 (70–93)	61 (45–76)	61 (43–76)	63 (46–78)
PPV	69 (48–86)	64 (49–78)	68 (53–81)	71 (56–83)
NPV	71 (57–82)	81 (63–93)	88 (70–98)	96 (80–100)
Accuracy	70 (59–80)	71 (60–81)	75 (64–85)	66 (54–77)
Evaluable patients (*n*)	84	76	73	73

CA125=cancer antigen 125; CR=complete response; PR=partial response; WHO=World Health Organization. Numbers in percentage (95% confidence intervals). CA125 ratio was calculated as the CA125 value after *M* (*M*=1–4) cycles divided by the baseline CA125 value.

demonstrates the sensitivity, specificity, PPV, NPV and concordance of the CA125 ratio response criteria in predicting a tumour response by WHO criteria (*n*=84). Of the four ratio criteria examined, the ratio criteria after three cycles of chemotherapy were found to provide the highest accuracy in predicting a tumour response by WHO criteria (75%; 95% CI: 64–85%). The reduction in accuracy after the third (75%) and the fourth (66%) cycle was not statistically significant (*P*=0.27).

## DISCUSSION

Several studies have compared CA125 response criteria with the WHO tumour response criteria in pretreated ovarian carcinoma patients receiving antineoplastic agents (Davelaar *et al*, 1996; Rustin *et al*, 1997; Bridgewater *et al*, 1999; Dieras *et al*, 2002) (Table 5

**Table 5:** Concordance between CA125 and WHO tumour response criteria in pretreated ovarian carcinoma

**Author**	**Year**	**No.**	**Treatment**	**Concordance (%)**
Davelaar	1996	77	Paclitaxel	30
Rustin	1997	72	Altretamin po	85
Bridgewater	1999	214	Paclitaxel	80
Bridgewater	1999	260	Platinum	73
Dieras	2002	27	Oxaliplatin	78
Gronlund	2004	124	Topotecan or Paclitaxel-Carboplatin	79

CA125=cancer antigen 125; WHO=World Health Organization. Concordance means that the designations of response or nonresponse were similar using CA125 and WHO tumour response criteria.

). Based on data from six different trials, Bridgewater *et al* (1999) found a high concordance between CA125 and WHO response criteria in patients treated with paclitaxel (80%) or platinum (73%). A high concordance was also found in patients treated with altretamin (85%) (Rustin *et al*, 1997) and oxaliplatin (78%) (Dieras *et al*, 2002). However, in a large study of Eisenhauer *et al* (1994), changes in CA125 levels were not always predictive of tumour response in paclitaxel treatment of 382 patients. Overall, 49% of patients demonstrated serial decreases in CA125 levels, but only one-third of these showed a response by standard tumour measurement criteria. Furthermore, five of 63 patients with increasing CA125 levels actually had objective tumour responses by standard criteria rather than disease progression. Unfortunately, the data in that study did not allow a calculation of concordance between CA125 and WHO response criteria. Similarly, Davelaar *et al* (1996) found a poor correlation between CA125 and WHO response criteria (30%), and concluded that CA125 does not seem to have any predictive value as to tumour response under paclitaxel treatment. It has been speculated that paclitaxel interferes with the interpretation of CA125 results because of increased shedding of CA125 antigen induced by paclitaxel, but this explanation has not been proved (Pearl *et al*, 1994). Also, in a study of 5FU-leucovorin in heavily pretreated patients, the change in CA125 levels failed to correlate with measured progression in one-third of the patients (Morgan *et al*, 1995). The results from the different studies have thus been conflicting, which may partly be explained by the fact that different CA125 assays and different CA125 cut-off levels have been applied, in contrast to the present study in which only one CA125 assay was used (Abbott CA125 EIA), and all serum CA125 analyses were analysed at the same laboratory (Statens Serum Institute). In a recent meta-analysis including 14 different drugs in 25 treatment groups, Rustin *et al* (2000) found that the CA125 and the WHO criteria were concordant in 20 of 25 groups, but overall the CA125 response rates were slightly higher than the WHO response rates by a factor 1.11.

In the present study, the GCIG CA125 response criteria significantly overestimated a documented decrease in tumour masses assessed by WHO criteria by 46% (*P*=0.045). Alternatively, the WHO response criteria underestimated a CA125 response by a factor 0.68. It is noted that patients with insufficient samples due to early progression were registered as nonevaluable (CA125 criteria), and this may represent a potential bias. Therefore, we performed an intent-to-treat analysis also including one patient with two elevated pretreatment samples and insufficient samples to be assessable by GCIG criteria, as suggested by Rustin (2003). In this analysis, the difference between WHO and GCIG CA125 response rates only tended to significance (*P*=0.055).

There are various reasons to explain the higher response rates of the CA125 criteria compared with a response by imaging-based methods such as the WHO (Miller *et al*, 1981), ECOG (Oken *et al*, 1982), GOG (Blessing, 1990) or the EORTC (Van Osterum, 1992) tumour response criteria. Recurrent ovarian carcinoma often spread to peritoneal surfaces forming multiple small nodules that may be difficult to assess with conventional imaging techniques (ultrasonography, CT scans or X-ray). It can be questioned as to how well the indicator lesions reflect overall tumour load. A chemotherapy-induced stabilisation of the indicator lesions may be registered simultaneously with considerable regression of diffuse carcinosis that is not measurable by WHO tumour response criteria and therefore not included in the overall evaluation of the tumour response. Alterations in the CA125 level may thus better reflect the total tumour load in ovarian carcinoma patients.

The overestimation of response by the GCIG CA125 criteria could partly be explained by the 14 patients being recorded as having SD (WHO response: nonresponders) but responding by CA125, but this patient group does not encompass all cases with discordant response designation.

In the present study, a high proportion of patients (83%) were assessable by WHO tumour response criteria, which may be due to the fact that all ultrasonographies were performed by specialists in Radiography. This facility is not available in all clinics, where assessment may be performed by general oncologists with considerably less experience. It is speculated that specialists in imaging methods may be able to detect tumour progression not detectable by a general oncologist thus increasing the rate of nonresponders, but this theory needs to be validated. Objective assessment methods such as the tumour marker CA125 may reduce the intra- and interobserver variability in the evaluation of patients with recurrent ovarian carcinoma.

It is important to rely on accurate response criteria in order to decide whether or not to continue a second-line chemotherapy that is considered as palliative. If, theoretically, the CA125 criteria overestimate a true therapeutic benefit as reflected by a response by WHO criteria, patients with chemo-resistant disease may continue an antineoplastic treatment without benefit in terms of tumour shrinkage, but with the potential risk of developing chemotherapy-induced toxicity. Similarly, if the WHO response criteria underestimate a true benefit of an antineoplastic regimen in terms of a reduction in the number of CA125-expressing cancer cells as reflected by a CA125 response, a potential active regimen may be prematurely withdrawn in preference to supportive care. In this study, none of the patients (0%) experienced a CA125 response despite a clinical disease progression, which means that none of the patients would have continued treatment despite tumour progression if CA125 criteria rather than WHO criteria had been used for guidance of second-line therapy. This finding is in agreement with other studies finding false-positive results of declining CA125 levels in very few patients (3%) (Bridgewater *et al*, 1999). These findings suggest that CA125 might be a more sensitive indicator of drug activity than WHO criteria. Failure of the CA125 response criteria to reflect a tumour shrinkage assessed by WHO criteria was observed in one patient, so the risk of discontinuing treatment on the basis of absence of CA125 response alone is low. In all, 21% of patients had diverging changes in tumour load and CA125 level. Patients with tumour shrinkage and a rising CA125 level may represent progression in resistant tumour clones as CA125-defined progression frequently antedates clinical progression by several months. Otherwise, a cancerous mass expressing high levels of CA125 may respond to treatment while part of the tumour with low expression of CA125 may exhibit progression.

Several definitions for CA125 alterations in the monitoring of ovarian carcinoma treatment have been proposed including single measurement of CA125 values (Davelaar *et al*, 1996), relative percentage reduction in the CA125 level (Eisenhauer *et al*, 1994; Rustin *et al*, 1996, 1997, 2000; Bridgewater *et al*, 1999; Campos *et al*, 2001; Meyer *et al*, 2001; Dieras *et al*, 2002; Rustin, 2003), CA125 ratio at selected time intervals (Davelaar *et al*, 1996; de Jong *et al*, 1997) and exponential regression analysis of the CA125 levels (Yedema *et al*, 1993; Pearl *et al*, 1994; de Jong *et al*, 1997). The only definition that has been validated prospectively in different data sets is the relative percentage response definition described by Rustin *et al* (1996). Generally, to be reliable enough for use in clinical trials, a CA125 response definition needs to take into account the natural variations in CA125 levels, upper limits of normal and missing samples. These demands are all met by the established Rustin CA125 criteria (50 and 75%), which uses mathematical logics for the systematic analysis of data, using a computer program (Rustin *et al*, 1999). However, second-line treatment of the individual patient with epithelial ovarian carcinoma represents another clinical setting where the main focus is palliation. Therefore, the simplicity of the selected CA125 criteria should be in focus in the clinical management of this patient segment, and accordingly, the simplified GCIG CA125 criteria seem attractive in clinics where computer-aided CA125 analyses are not yet available. Irrespective of the preferred CA125 response algorithm, serial measurement of CA125 is an important tool in the clinical decision making, especially in patients with ill-defined tumours.

Using an alternative CA125 response algorithm (CA125 ratio), the highest accuracy in predicting a tumour response by WHO criteria was noted after three cycles of chemotherapy (75%). The accuracy after the fourth cycle (66%) was numerically inferior. This may be explained by the advent of early third relapses verified by the finding in 54 patients with initial response after two cycles, in which five patients (9%) experienced a subsequent progression in CA125 levels after three or four cycles. Furthermore, the fluctuation in CA125 levels may have an impact on the comparison of CA125 ratios after different number of treatment cycles, because the CA125 ratio response definitions do not require a confirmatory sample. In summary, in terms of accuracy the ratio criteria added little compared to the GCIG CA125 response criteria selected in this study as the main CA125 response algorithm. However, wide ranges in CIs were noted in all CA125 algorithms.

In conclusion, the GCIG CA125 criteria overestimate a tumour response measured by WHO criteria in monitoring the efficacy of topotecan or paclitaxel–carboplatin when used as second-line regimens in patients with epithelial ovarian carcinoma. Secondly, the accuracy of CA125-based response criteria in predicting a tumour response by WHO criteria was not increased by using CA125 ratio criteria. In patients with diverging changes in response assessed by imaging-based methods or in tumour marker levels, other parameters such as improvements in symptoms should be emphasised to guide treatment. Future studies will reveal if imaging-based criteria or CA125-based response criteria best reflect the efficacy of second-line chemotherapy as related to survival.
